# Calcitonin gene-related peptide stimulates proliferation of alveolar epithelial cells

**DOI:** 10.1186/1465-9921-10-8

**Published:** 2009-02-03

**Authors:** Yukiko Kawanami, Yasuo Morimoto, Heungnam Kim, Takehiro Nakamura, Kazuhiko Machida, Takashi Kido, Etsuko Asonuma, Kazuhiro Yatera, Chiharu Yoshii, Masamitsu Kido

**Affiliations:** 1 Department of Respiratory Disease, University of Occupational and Environmental Health, Japan, Kitakyushu City, Fukuoka, Japan; 2 Department of Occupational Pneumology, University of Occupational and Environmental Health, Japan, Kitakyushu City, Fukuoka, Japan; 3 Department of Environmental Health Engineering, University of Occupational and Environmental Health, Japan, Kitakyushu City, Fukuoka, Japan

## Abstract

**Background:**

Alveolar epithelial cells are known as progenitor cells for the restoration from the damage in the lung. Calcitonin gene-related peptide (CGRP) has been reported to play an important role in the proliferation of various types of epithelial and endothelial cells. We investigated the effects of CGRP on the proliferation of alveolar epithelial cells *in vitro *and *in vivo*.

**Methods:**

A549 cells were cultured in Dulbecco Modified Eagle Medium with 5% fatal bovin serum for 24 hours, then CGRP was added *in vitro*. The proliferation of DNA synthesis was measured using 5-bromo-2-deoxyuridine, an analog of thymidine, by enzyme-linked immunosorbent assay.

As one intracellular response to CGRP, we examined activation of p44/42- extracellular signal-regulated kinase (ERK) pathway by adding CGRP, using western blotting method.

Recombinant adenovirus encoding nuclear-targeted-human β-CGRP (rhCGRP) was administered into Male Wister rat (n = 5, 10 weeks old) lungs by intratracheal instillation *in vivo*. 7 days after the administration of CGRP, rat lungs were harvested and histological findings and immunohistochemical staining of proliferating cell nuclear antigen (PCNA) were evaluated to examine cell proliferation.

**Results:**

*In vitro *study, CGRP increased the proliferation of A549 cells in a dose and time dependent manner. CGRP8-37 (inhibitor of CGRP receptor) decreased CGRP induced proliferation of DNA synthesis. Phosphorylation of ERK pathway was observed within 15 minutes and peaked in one hour. U0126 (inhibitor of ERK pathway) decreased CGRP induced proliferation of DNA synthesis.*In vivo *study, histological examination of the lung indicated proliferation of alveolar epithelial cells in the rhCGRP-treated group and the nuclei of alveolar epithelial cells were positive for PCNA immunostaining.

**Conclusion:**

In this study, we conclude that CGRP stimulates proliferation of human alveolar epithelial cells *in vivo *and *in vitro*.

## Background

Organisms innately possess the ability to promptly repair the injury inflicted upon the individual as one of the defence mechanisms so that they can maintain the normal functions and structures of tissues and organs. After lung injury, alveolar epithelial type II cells are known as progenitor cells in the lung to regenerate and restore the damage. Accordingly, it is hypothesized that factors which facilitate proliferation of these cells would contribute to the enhancement of lung regeneration and furthermore may lead to treatment option.

Calcitonin gene-related peptide (CGRP) is 37-amino acid neuropeptide found among neuroendocrine cells, sensory C fibers, blood vesssels and lymphoid tissues in the normal lung [1,2]. In addition to the function of vasodilatation [3], bronchial protection [4], regulatory function of macrophages [5] and regulation of airway responsiveness [6], CGRP has been reported to play a role in proliferation of epithelial and endothelial cells of various types, such as human endothelial cells [7], tracheal epithelial cells [8,9], retinal pigment epithelial cells [10] and thymic epithelial cells [11].

Meanwhile, it has been reported that pulmonary neuroendocrine cells and neuroendocrine bodies become neuroendocrine cell hyperplasia during the restoration process, and these cells produce CGRP and have paracrine effect on cell proliferation in the lung microenvironment in airway and alveolar epithelial injury animal models [12]. We have reported that relation between inflammation or fibrosis and CGRP expression in the animal models exposed to dusts of various types [13]. In the models of lung injury induced by crystalline silica, crocidolite asbestos and bleomycin, pathological findings of lung inflammation increased progressively in a time dependent manner, while low levels of CGRP concentration in lungs were observed [13]. On the other hand, in lung injury induced by exposure to silicon carbide whisker and potassium octatitanate whisker, strong inflammation was transient, then lung inflammation abated gradually, while high levels of CGRP concentration in the lung were observed at an early phase [13]. These findings suggest that CGRP is involved in the lung restoration. Moreover, it has been reported that ablation of the CGRP-releasing sensory neurons aggravates severe inflammation, and absence of CGRP intensifies the inflammatory reaction of tissues induced by reperfusion in the CGRP-knockout mice [14]. It is therefore thought that CGRP and sensory neurons play an important role in maintaining the tissue integrity by regulating local inflammatory responses in the lung.

The extracellular signal-regulated kinase (ERK) pathway is a critical pathway involved in the proliferation of many cell types including alveolar epithelial cells.

On the basis of our hypothesis that CGRP proliferates alveolar epithelial cells, we have examined the effects of CGRP on the proliferation of alveolar epithelial cells and ERK signalling pathway as one of the cell proliferation pathways *in vitro *and *in vivo*.

## Methods

### Cell culture

A549 cells (RIKEN gene bank, Japan), originally derived from human lung carcinoma were cultured in Dulbecco Modified Eagle Medium (GIBCO Invitrogen, Japan) with 5% fatal bovin serum (FBS) (Biosource, Japan) containing 0.1% penicillin/streptomycin (Nakarai, Japan) in a humidified incubator with 5% CO_2 _at 37°C. Cell cultures were passaged at confluency approximately every three days. Cells were then removed from monolayer culture in trypsin containing ethylinedimine tetraacetic acid, counted with a hemocytometer and plated in each well. After incubating at 37°C for 24 hours, the cells were washed with phosphate buffered saline (PBS) and then cultured with Human CGRP (PEPTIDE INSTITUTE, Japan) at variable concentrations for different time periods.

### Cell proliferation analysis

In order to evaluate the effect of CGRP on proliferation of A549 cells, DNA of proliferating cells were measured by colorimetric 5-bromo-2-deoxyuridine (BrdU) Cell Proliferation ELISA Kit (Roche, Japan). A549 cells were plated in 96-well dishes (32 mm^2^/well, 6.0 × 10^3 ^cells/well) with a medium volume of 100 μl/well. After 24 hours of incubation, the cells were treated with 100 μl of 10^-13 ^to 10^-8 ^M CGRP and incubated for 12, 24 and 36 hours. The cells were reincubated for additional 2 hours at 37°C by adding BrdU labelling solution (10 μl/well, final concentration: 10 μM), after which the labelling medium was drained. The cells were dried with a hair-dryer for about 15 minutes and stored at 4°C for a few days. FixDenat solution (200 μl/well) was added into each well and incubated for 30 minutes at room temperature. FixDenat solution was immediately and thoroughly drained and anti-BrdU-peroxidase working solution (100 μl/well) was added and incubated for 30 minutes at room temperature. Antibody conjugate was immediately removed and the wells were rinsed three times with washing solution (200–300 μl/well). Substrate solution (100 μl/ml) was added and incubated at room temperature for about 5 minutes until the colour development is sufficient for photometric detection. Absorbance was measured using an automatic enzyme-linked immunosorbent assay (ELISA) reader (570 nm). The data was shown as a percentage for the control.

In order to examine the influence of CGRP receptor and ERK pathway, proliferation of A549 cells was measured by the same method 24 hours after adding CGRP (10^-10 ^M) and each inhibitor, U0126 (1 μM, inhibitor of ERK pathway, Promega, USA) and CGRP8-37 (10^-8 ^M, CGRP-receptor antagonist, Sigma Chemical Co., USA).

### Measurement of ERK phosphorylation (Western blot analysis)

A549 cells were plated in 6-well dishes (962 mm^2^/well, 1.5 × 10^5 ^cells/well) with a medium volume of 2 ml/well. After adding CGRP, the cells were incubated for 24 hours. Cell pellets were collected and membrane protein was isolated by using ReadyPrep Protein Extraction Kit (Biorad, USA). Protein levels were determined using the RC-DC Assay Kit (Biorad). Aliquots of 20–30 μg protein were boiled in loading buffer (New England Biolabs, USA) for 2 minutes, then loaded onto 12% Tris-glycine-polyacrylamide gel (Cambrex, USA) and transferred electrophoretically to nitrocellulose membranes.

40 μg of cell homogenate protein were added to gel loading buffer (final concentration: 50 mM Tris, 70 mM NaCl, 1% sodium lauryl sulfate (SDS), 5% 2-mercaptoethanol) and electrophoresed on polyacrylamide gel (15%) in the presence of 0.1% SDS by using the Mini Protein 3 Dlectrophoresis System (Bio-Rad). The samples were electrophoretically blotted on polyvinylidine difloride membrane (ECL Western Blotting Detection System, Amersham Biosciences, USA) at 25V for one hour by transblot apparatus. Subsequently, the samples were incubated with 3% milk-Tris-buffered saline-Tween 20 for one hour, after which they were incubated at room temperature overnight with rabbit anti-human p42/44 ERK and phospho- p42/44 ERK antibody (dilution 1/1,000, Cell Signalling Technology Inc., Japan) in 3% milk-Tris-buffered saline-Tween 20. After three times of washing, the membrane was incubated with peroxidase-conjugated sheep anti-rabbit IgG (Amersham Biosciences) (dilution 1/5,000) for one hour. The membrane was then washed three times, and the signals were finally visualized using the enhanced chemiluminescence system (Amersham Biosciences). The bands of p42/44 ERK and phospho- p42/44 ERK were analyzed with the National Institutes of Health Image 1.56 software (written by Wane Rasband at NIH, Bethesda, USA).

### Recombinant human CGRP (rhCGRP) Treatment

Recombinant adenovirus encoding nuclear-targeted-human β-CGRP was generated using Adeno-X Expression System 1 (Clontech Laboratories, USA). First, a mammalian expression cassette was made by cloning human β-CGRP gene (ResGen, USA) into pShuttle2. After amplification in *E. coli*, the expression cassette was excised from pShuttle2 and ligated to Adeno-X Viral DNA (the adenoviral genome). Because pShuttle2 and Adeno-X Viral DNA carried different antibiotic selection markers, we did not purify the expression cassette fragment for ligation with Adeno-X. In the final stage, the recombinant Adeno-X vector was packaged into infectious adenovirus by transfecting human embryonic kidney 293 cells. Recombinant adenovirus was harvested by lysing transfected cells. Recombinant adenoviruses titer was determined by Titer Kit (Clontech Laboratories). Purified viruses were stored in PBS and kept at -80°C until use.

### In vivo analysis

The rats were handled according to the guidelines described in the Japanese Guide for the Care and Use of Laboratory Animals as approved by the University of Occupational and Environmental Health Animal Care and Use Committee. Male Wister rats (n = 5, 10 weeks old, mean body weight 256 ± 20 g) were purchased from Kyudo (Kumamoto, Japan). They were anesthetized with inhaled ether in the box, and treated by intratracheal injection of rhCGRP (4.0 × 10^8 ^infectious unit) or saline through a cannula using a laryngoscope. They were assigned to a rhCGRP group (n = 5) and a saline control group (n = 5) for each exposure category and maintained for 7 days after the injection. Each rat was then sacrificed with a fatal over dosage of Nembutal (Dainippon Sumitomo Pharma, Japan) intraperitoneally. After severing the abdominal aorta, both lungs were removed and fixed by intratracheal instillation of 4% paraformaldehyde (MERCK, Japan) at 25 cmH_2_O pressure. The lung and trachea were excised from the surrounding tissue and allowed to stand at 4°C for 24 hours. The tissue was washed for 10 minutes in PBS, dehydrated by immersing in a graded series of one hour ethanol washes, before being finally maintained in 100% ethanol at 4°C. The lung tissue was embedded in paraffin and cut into each 5 μm section, and Hematoxylin-Eosin (HE) staining and immunohistochemical staining for proliferating cell nuclear antigen (PCNA) (Dako, Denmark) were performed.

For PCNA immunohistochemistry, the tissue sections were air-dried, and deparaffinized by washing in xylene for 3 minutes four times, followed by dehydration through a series of 100% to 70% ethanol washes. The slides were placed in methanol containing 0.3% hydrogen peroxide to block endogenous peroxidase activity. Nonspecific binding was blocked by incubating with 10% goat serum for 10 minutes at room temperature. Then the lung sections were incubated overnight at 4°C with monoclonal mouse antibody specific for PCNA (diluted 1:500–1:1000 before use). The sections were rinsed three times in 0.1 M PBS and incubated for 30 minutes at room temperature with biotinylated anti-mouse secondary antibody, then incubated with avidin-biotin complex (DAKO JAPAN, Japan) for 30 minutes at room temperature. Sections were washed with PBS and incubated with diaminobenzidine for 7 minutes, and counterstained with hematoxylin. Tissue incubations without the primary or secondary antibody were carried out as labeling controls.

### Statistical analysis

Values were expressed as mean ± one standard deviation. We used non-parametric Mann-Whitney U test for the statistical analysis with two groups. Statistical significance was analyzed using analysis of variance. A probability (*p*) value less than 0.05 was considered significant.

## Results

### Cell proliferation by CGRP in vitro

**Figure 1:**
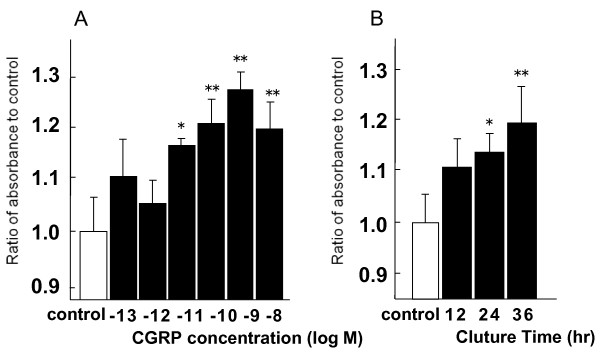
**BrdU incorporation induced by CGRP on A549 cells**. CGRP increased BrdU incorporation in a dose dependent manner (A) and a time dependent manner (B). Cells were prepared as described in Methods. Data represents the means ± SEM of five experiments. *: p < 0.05 compared to control **: p < 0.01 compared to control.

CGRP (10^-11^-10^-8 ^M) significantly increased the BrdU incorporation in A549 cells in a dose dependent manner (Fig. 1A). Similar tendency was observed at 12, 24 and 36 hours after the exposure. CGRP-induced increase of BrdU incorporation was also observed in a time dependent manner (Fig. 1B).

**Figure 2:**
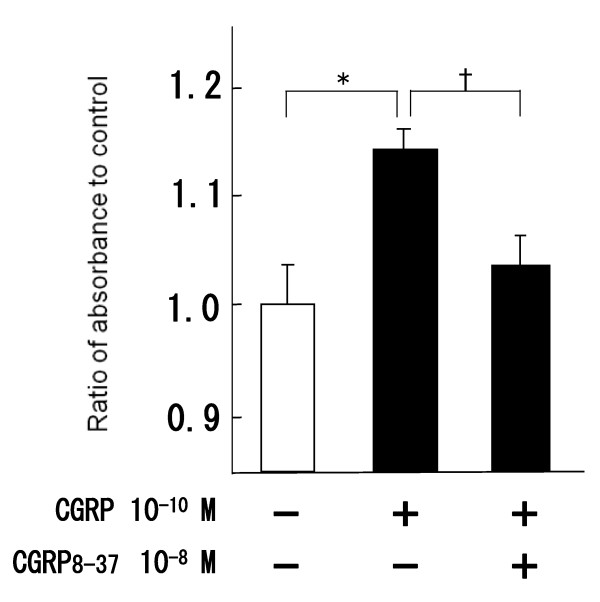
**Inhibition of BrdU incorporation induced by CGRP receptor antagonist**. CGRP8-37 (inhibitor of CGRP receptor, 10^-8 ^M) decreased CGRP-induced proliferation. *: P < 0.05 compared to control †: p < 0.05 compared to only CGRP 10^-10 ^M.

Compared to the group treated with CGRP only, preadministration of CGRP-receptor antagonist (CGRP 8–37) significantly inhibited the increase of BrdU incorporation (Fig. 2).

### ERK phosphorylation in vitro

**Figure 3:**
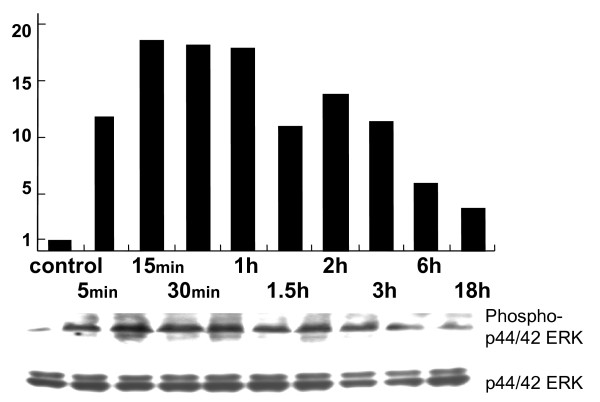
**Western blotting of p44/42- extracellular signal-regulated kinase**. Activation of p44/42-ERK pathway by adding CGRP occurred within 15 minutes and peaked in one hour.

p42/44-ERK protein was phosphorylated with tyrosine and threonine within 15 minutes, and the phosphorylation maintained its maximal effects for one hour (Fig. 3).

These data suggest that CGRP stimulates the proliferation of human alveolar epithelial cells via ERK pathway.

**Figure 4:**
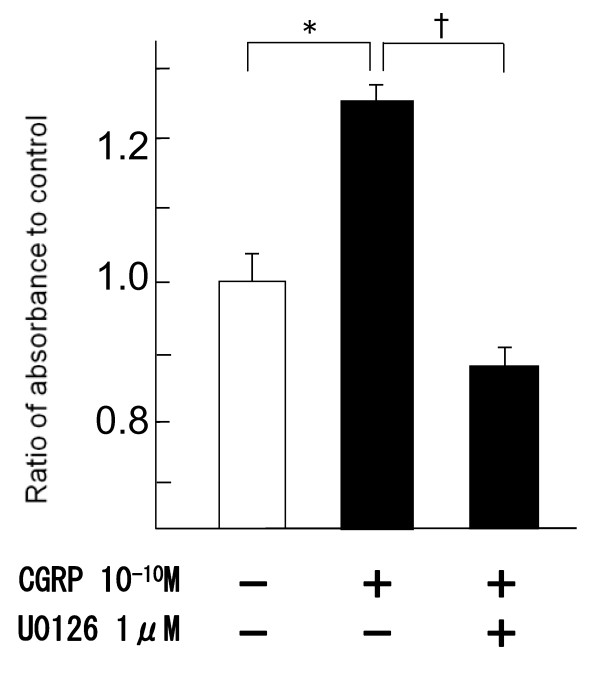
**Inhibition of BrdU incorporation induced by ERK inhibitor**. U0126 (inhibitor of ERK pathway, 10^-6 ^M) decreased the CGRP-induced proliferation. *: p < 0.05 compared to control †: p < 0.01 compared to only CGRP10^-10 ^M.

Compared to the group with CGRP only, addition of CGRP and ERK inhibitor (U0126) significantly inhibited the increase of BrdU incorporation (Fig. 4).

### Effect of recombinant human CGRP vector in rat lung

**Figure 5:**
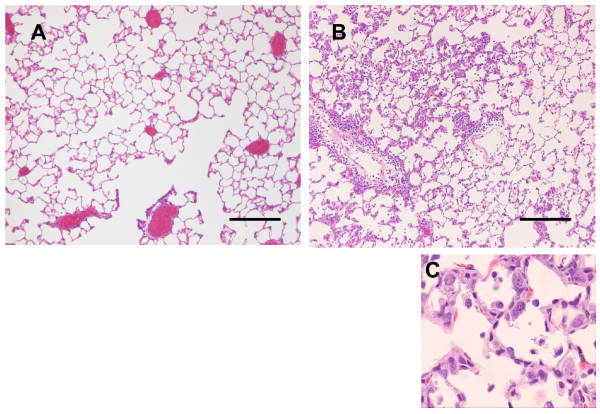
**Histopathology of CGRP treated rat lung**. Recombinant human CGRP (rhCGRP) were administered into Wister rat lung by intratracheal instillation. Proliferation of alveolar epithelial cells and interstitial thickening were observed in the rhCGRP-exposed group (B, C) compared with those in the control group (A) 7 days after CGRP exposure. The bars represent 200 μm.

**Figure 6:**
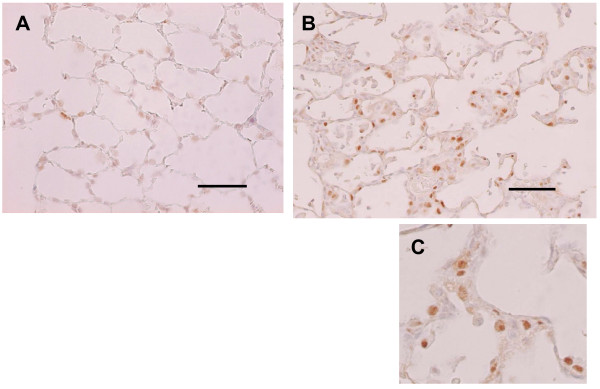
**Immunohistochemistry for Proliferating Cell Nuclear Antigen**. Histological examination indicated proliferation of alveolar epithelial cells, and PCNA immunohistochemical staining represented that the nuclei of these cells were positive in the group administered rhCGRP (B, C), and few positive nuclei were observed in the control group (A). The bars represent 100 μm.

Proliferation of alveolar epithelial cells and interstitial thickening were observed in the rhCGRP-exposed group compared with the control group (Fig. 5). Similarly, proliferation of bronchial epithelial cells was observed while mild interstitial infiltration of lymphocytes was also observed. Immunostaining for PCNA was observed in the nuclei of alveolar epithelial cells (Fig. 6).

## Discussion

In this study, we observed that CGRP facilitates proliferation of A549 cells via CGRP receptor and ERK pathway *in vitro*.

Regarding the proliferative effects of CGRP on various epithelial and endothelial cells, CGRP has been reported to proliferate human endothelial cells [7], airway epithelial cells [8,9] retinal pigment epithelial cells [10], thymic epithelial cells [11]. On the other hand, no such effects have been indicated on lung fibroblast cells [15] and inhibitory function has been found on vascular smooth muscle cells [16].

In the present study, 10 pM-10 nM CGRP significantly increased A549 cell proliferation. Other studies conducted with guinea pig tracheal epithelial cells, retinal pigment epithelial cell or rat thymic epithelial cells, 1 pM-1 μM CGRP have also showed cell proliferation, and our data fell within this range [7-11]. CGRP concentration in the rat lung was 18.5 nM, and the exposure level of the concentration in this study was considered as physiological.

It has also been reported that growth factors, such as epidermal growth factor, hepatocyte growth factor (HGF) and keratinocyte growth factor (KGF) have proliferative effect on alveolar epithelial cells [17-19]. According to these previous reports, HGF increases thymidine incorporation 3.4 times as much, compared to the control group [17], whereas recombinant adenovirus vector expressing KGF (Ad5-KGF) increases thymidine incorporation in a dose dependent manner [18]. In our experiment, effect of CGRP was as minor as approximately 1.2 to 1.3 times compared to other growth factors. In comparative studies on the effects of HGF and KGF, KGF seemed to have larger proliferative effect on alveolar epithelial cells *in vitro *but the contrary effects were observed *in vivo *[20,21]. As is suggested by these results, comparison between proliferation of alveolar epithelial cells *in vivo *and *in vitro *cannot be generalized.

In the present *in vivo *experiment, proliferation of alveolar epithelial cells was actually observed in the rat lung in which CGRP expression was artificially created. It was reported that proliferation of alveolar epithelial cells peaked two days after the exposure of HGF intratracheally in the rat lung [22]. Meanwhile, hyperplasia of alveolar epithelial cells reportedly occurred in the early phase (2–7 days after the exposure) in the rat lungs dissected 2–28 days after KGF intratracheal injection [18]. The finding of alveolar epithelial cell proliferation on the seventh day after the CGRP exposure is similar to other growth factors.

The result that preadministration of CGRP receptor inhibitor restrained increase of alveolar epithelial cell proliferation by CGRP seems to be supported by the previous findings that CGRP was produced by the paracrine effect in the lung microenvironment, resulting in proliferation of airway and alveolar epithelial cells [12] and that CGRP receptors were found in the membrane of A549 cells [23].

Generally, CGRP in the normal lung is secreted from neuroendocrine cells existing in the central airways [24], and there are several reports indicating that CGRP is secreted from A549 cells [23,25]. In these reports, CGRP secretion from A549 cells increased under the condition of hypoxia, and they also suggested that distribution of CGRP secretion would be different under stressful situations.

We also observed ERK phosphorylation during CGRP induced cell proliferation. The ERK pathway is a critical pathway involved in the proliferation of many cell types, and proliferation of alveolar epithelial cells induced by ERK activation has been reported [26]. Our result showed that ERK activation peaked between 5 minutes and one hour after the exposure of CGRP and continued for at least 18 hours. In studies with KGF which proliferates alveolar epithelial cells, ERK activation appeared after 5 minutes and lasted for 90 minutes [27]. Such result showed similar tendency to the findings in this study.

Harada *et al. *reported that CGRP increased insulin-like growth factor-1 (IGF-1) production [28], so we also examined pulmonary immunohistochemical staining of IGF-1 in this study. No significantly immunostained for IGF-1 cells were observed in the lung in CGRP exposed group, and IGF-1 levels in culture medium of A549 cells after exposure to CGRP did not increase. Therefore we think that IGF-1 induced by CGRP may be minimal in the rat lung.

Recent reports suggest that growth factors, such as HGF and KGF, proliferate alveolar epithelial cells while they inhibit lung injury. Increased function of cell proliferation *in vivo *and *in vitro *is thought to be involved with lung restoration. We would like to hereafter examine the effects of CGRP preadministration in various types of lung injury models.

In conclusion, this study describes the proliferative effects of CGRP on A549 cells in a dose dependent and time dependent manner *in vitro*, via CGRP receptor and ERK pathway. Proliferation of alveolar epithelial cells and interstitial thickening were also observed in CGRP exposed rat lung *in vivo*, and we speculate that CGRP has proliferatory function on alveolar epithelial cells.

## Competing interests

The authors declare that they have no competing interests.

## Authors' contributions

YK, YM, KH, KY, CY and MK designed of the study. YK played a major role in the acquisition, analysis and interpretation of data and drafted the manuscript. KH made CGRP-adenovirus vector. YK, YM, KH, TN, KM, TK, and EA participated in the *in vivo *procedures, and carried out the challenge. KH performed the immunohistochemistry. All authors read and approved the final manuscript.
